# Serum γ-glutamyl Transferase Levels, Insulin Resistance and Liver Fibrosis in Patients with Chronic Liver Diseases

**DOI:** 10.1371/journal.pone.0051165

**Published:** 2012-12-05

**Authors:** Salvatore Petta, Fabio Salvatore Macaluso, Maria Rosa Barcellona, Calogero Cammà, Daniela Cabibi, Vito Di Marco, Antonio Craxì

**Affiliations:** 1 Sezione di Gastroenterologia, DiBiMIS, University of Palermo, Palermo, Italy; 2 Cattedra di Anatomia Patologica, University of Palermo, Palermo, Italy; Yonsei University College of Medicine, Republic of Korea

## Abstract

**Background and Aims:**

Serum levels of γ-glutamyl-transpeptidase(γ-GT) were associated with liver disease severity and metabolic alterations, which in turn are able to affect hepatic damage. In patients with nonalcoholic fatty liver disease (NAFLD), genotype 1 chronic hepatitis C (G1CHC) and chronic hepatitis B (CHB), we assessed the link between liver fibrosis and γ-GT serum levels, and we evaluated if normal or high γ-GT serum levels affect the association between insulin resistance (IR) and severity of liver fibrosis.

**Methods:**

843 consecutive patients with chronic liver disease (CLD)(193 NAFLD, 481 G1CHC, 169 CHB) were evaluated by liver biopsy (Kleiner and Scheuer scores) and clinical and metabolic measurements. IR was diagnosed if HOMA>3. A serum γ-GT concentration of >36 IU/L in females and >61 IU/L in males was considered the threshold value for identifying high levels of γ-GT.

**Results:**

By multivariate logistic regression analysis, abnormal γ-GT serum levels were independently linked to severe liver fibrosis in patients with NAFLD (OR2.711,CI1.120–6.564,p = 0.02), G1CHC (OR3.461,CI2.138–5.603,p<0.001) and CHB (OR2.778,CI1.042–7.414,p = 0.04), together with IR and liver necroinflammation, and with a negative predictive value>80%. Interestingly, among patients with high or normal γ-GT values, even if IR prevalence was significantly higher in patients with severe fibrosis compared to those without, IR remained significantly associated with severe fibrosis in patients with abnormal γ-GT values only (OR4.150,CI1.079–15.970,p = 0.03 for NAFLD; OR2.250,CI1.211–4.181,p = 0.01 for G1CHC; OR3.096,CI2.050–34.220,p = 0.01 for CHB).

**Conclusions:**

In patients with CLD, IR is independently linked to liver fibrosis only in patients with abnormal γ-GT values, without differences according to liver disease etiology, and suggesting a role of γ-GT as a marker of metabolic-induced liver damage. These data could be useful for the clinical and pharmacologic management of patients with CLD.

## Introduction

The prognosis of patients with chronic liver diseases (CLD), independently of the underlying etiology, is ultimately decided by the amount of liver fibrosis that will accumulate over the years as a consequence of several mechanisms of tissue injury, with the ultimate development of cirrhosis and its complicances [1], [2]. Other than conventional host and viral factors known to affect liver fibrosis progression, metabolic factors, and especially insulin resistance (IR) act as major disease modifiers associated with the severity of hepatic damage. Specifically, IR is the key determinant of nonalcoholic fatty liver disease (NAFLD) and its severity [3], is common in western patients with chronic hepatitis C (CHC) [4], due to both viral and host factors [5], and associated with the severity of fibrosis and its progression independently of steatosis and visceral obesity [6], [7], and finally seems also associated with the severity of liver fibrosis in patients with chronic hepatitis B (CHB) [8].

However, nevertheless these data, it is probably that the impact of IR on the severity of liver fibrosis is different according to the clinical setting. These considerations in fact arise from contrasting evidences about the association between liver fibrosis and IR observed in some studies on patients with CHC [4], [9] or CHB [10], and from the evidences that insulin-sensitizer therapies obtain an improvement in histological outcomes in a proportion of NAFLD patients only [11], [12].

γ-glutamyl transpeptidase (γ-GT) is a hepatic and biliary enzyme synthesized by hepatocytes as well as epithelial cells of intra-hepatic bile ducts [13], [14]. Several cross-sectional and prospective studies showed that plasma γ-GT is associated with metabolic syndrome risk factors and with markers of inflammation [15]–[18], also representing in large population studies an independent predictor of all-cause, liver-related, diabetes-related, and cancer-related death [13]. In this line, recent studies suggest that plasma γ-GT is a significant predictor of NAFLD, independently of alcohol intake [14], [15], [19], and that more specifically elevated γ-GT levels are associated with a more severe histological spectrum of NAFLD, namely the presence of NASH and fibrosis [16], whereas reductions in γ-GT predict histological improvement in NAFLD following bariatric surgery [15], [20]. Similarly, different cross-sectional studies also showed that higher γ-GT serum levels are predictive of the severity of liver fibrosis in patients with both CHC and CHB, being this test also included in non-invasive scores for hepatic fibrosis assessment [21], [22].

All these data therefore could suggest that γ-GT is expression of an environmental and probably genetic state of inflammation and metabolic dysfunction, affecting the spectrum of the severity of liver disease.

In light of these facts, we aimed to confirm the association between severity of liver disease and γ-GT serum levels in patients with an histological diagnosis of CLD, due to NAFLD, CHC and CHB, and to evaluate, in these patients, if the presence of normal or high γ-GT serum levels affects the association between IR and severity of liver fibrosis.

## Materials and Methods

### Patients

Eight-hundred and forty-three consecutive patients with CLD, due to viral or metabolic etiology (193 with NAFLD, 481 with genotype 1 CHC, and 169 with CHB), recruited at the Gastrointestinal & Liver Unit at the University Hospital in Palermo and fulfilling all inclusion and exclusion criteria detailed below, were assessed. (a) histological diagnosis of NAFLD, or G1 CHC, or CHB on a liver biopsy performed less than 6 months before enrollment; (b) NAFLD diagnosed on the basis of chronically elevated ALT for at least 6 months, alcohol consumption of <20 g/day, steatosis (>5% of hepatocytes) at histology with necroinflammation and/or fibrosis, and negative anti-HCV and HBsAG; (c) G1 CHC characterized by the presence of anti-HCV and HCVRNA, with persistently abnormal alanine aminotransferase (ALT), alcohol consumption of <20 g/day in the last 12 months or more, naive to antiviral therapy, and negative HBsAg; (d) CHB characterized by the presence of HBsAg and HBVDNA >2000 IU/ml, with persistently abnormal alanine aminotransferase (ALT), alcohol consumption of <20 g/day in the last 12 months or more, naive to antiviral therapy, and negative anti-HCV. Exclusion criteria were: (1) advanced cirrhosis; (2) hepatocellular carcinoma; (3) other causes of liver disease or mixed aetiologies; (4) human immunodeficiency virus infection; (5) previous treatment with antiviral therapy, immunosuppressive drugs and/or regular use of steatosis-inducing drugs, evaluated by interview; (6) active intravenous drug addiction.

**Figure 1:**
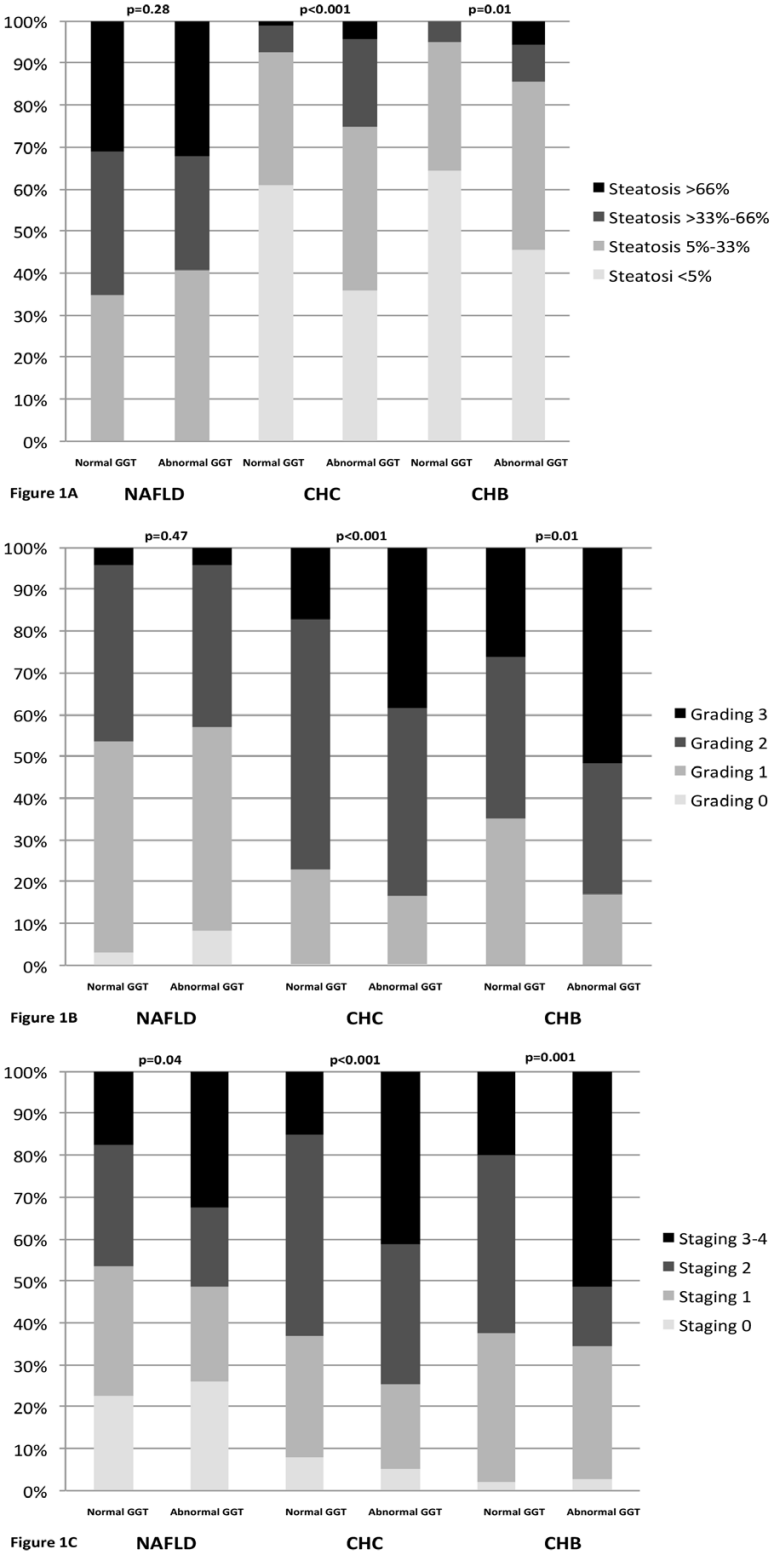
Histological characteristics of patients. (A) Steatosis grade according to liver disease etiology and normal/abnormal γ-GT values; (B) Grade of liver inflammation, according to liver disease etiology (Kleiner score for NAFLD, Scheuer score for CHC and CHB) and normal/abnormal γ-GT values; (C) Stage of liver fibrosis, according to liver disease etiology (Kleiner score for NAFLD, Scheuer score for CHC and CHB) and normal/abnormal γ-GT values.

**Table 1:** Demographic, Clinical, Biochemical, and Histological Features of 843 Patients with Metabolic and Viral Chronic Liver Diseases, According to the Etiology and to γ-glutamiltranspherase Serum Levels.

Variable	Non-alcoholic Fatty Liver Disease N = 193			Genotype 1 Chronic Hepatitis C N = 481			Chronic Hepatitis B N = 169		
	normal γGT (n = 97)	abnormal γGT (n = 96)	P value	normal γGT n = 273	abnormal γGT n = 208	P value	normal γGT n = 134	abnormal γGT n = 35	P value
**Mean age** – **yrs**	44.7±13.6	46.7±13.2	0.31	51.9±12.1	54.2±11.8	0.03	38.6±13.7	46.7±13.1	0.002
**Male Gender**	78	50	<0.001	131	111	0.24	102	21	0.05
**Mean body mass index** – **kg/m^2^**	30.0±4.5	29.5±4.8	0.42	26.2±4.2	26.7±4.1	0.22	25.4±3.2	26.0±3.3	0.37
**Alanine aminotransferase – IU/L**	70.0±56.7	89.2±52.9	0.01	61.7±38.2	128.4±128.8	<0.001	93.7±94.9	138.5±99.5	0.01
**γ-glutamiltranspherase –IU/L**	32.8±13.7	155.9±143.2	<0.001	27.5±13.4	135.4±155.5	<0.001	28.5±13.3	83.5±46.9	<0.001
**Cholesterol – mg/Dl**	192.8±42.0	217.0±49.9	<0.001	175.9±36.0	173.8±36.4	0.53	185.2±39.1	211.7±57.7	0.01
**Triglycerides – mg/dL**	136.4±69.8	161.9±83.1	0.02	88.2±40.0	107.2±49.1	<0.001	96.7±33.2	117.2±66.6	0.01
**Blood glucose – mg/dL**	97.4±23.0	101.1±37.8	0.41	92.9±25.4	103.7±42.3	0.001	88.5±16.8	98.0±28.5	0.01
**Insulin – µU/mL**	16.3±18.5	18.2±11.8	0.20	12.3±8.9	14.1±7.5	0.02	10.0±6.9	12.0±8.0	0.15
**HOMA-score**	4.05±2.95	4.73±3.93	0.18	2.97±2.23	3.56±2.64	0.001	2.21±1.60	3.02±2.37	0.01
**Insulin Resistance**	44	49	0.43	98	115	<0.001	29	13	0.05
**Type 2 diabetes**	17	16	0.84	29	40	0.008	3	3	0.07
Stage of Fibrosis*									
** 0**	22	25		22	11		7	1	
** 1**	30	22		79	42		46	11	
** 2**	28	18		131	69		55	5	
** 3**	15	20		29	48		18	8	
** 4**	2	11	0.04	12	38	<0.001	8	10	0.001

Data are given as mean ± SD or as number of cases. Yrs, indicates years; HOMA, homeostasis model assessment. * Kleiner score for non-alcoholic fatty liver disease patients, and Scheuer score for genotype 1 chronic hepatitis C, and chronic hepatitis B patients.

The study was carried out in accordance with the principles of the Declaration of Helsinki and its appendices, and with local and national laws. Approval was obtained from the hospital’s Internal Review Board and its Ethics Committee, and written informed consent was obtained from all patients and controls.

**Table 2:** Univariate and Multivariate Analysis of Risk Factors Associated with Severe Fibrosis (F3–F4) in 843 Patients with Metabolic and Viral Chronic Liver Diseases by Logistic Regression Analysis.

Variable	No Severe Fibrosis(F0–F2)	Severe Fibrosis(F3–F4)	Univariate Analysis *p* value	Multivariate Analysis OR (95% CI) *p* value
**Non-alcoholic Fatty Liver Disease**				
	**n = 144**	**n = 49**		
**Age** – **yrs**	42.9±12.6	54.0±12.3	<0.001	1.063 (1.028–1.100) <0.001
**Male Gender**	104	40	0.003	1.079 (0.457–2.546) 0.86
**Body Mass Index – Kg/m2**	29.3±4.7	31.1±4.2	0.01	1.044 (0.948–1.150) 0.37
**Abnormal γ-GT**	65	31	0.02	2.711 (1.120–6.564) 0.02
**Insulin Resistance**	57	36	<0.001	2.749 (1.153–6.556) 0.02
**Histology at Biopsy**				
** Lobular Inflammation 0/1/2/3**	11/84/46/3	0/12/32/5	<0.001	4.531 (2.244–9.147) <0.001
**Genotype 1 Chronic Hepatitis C**				
	**n = 354**	**n = 127**		
**Age** – **yrs**	51.1±12.3	57.8±9.5	<0.001	1.044 (1.022–1.067) <0.001
**Male Gender**	185	54	0.06	0.639 (0.397–1.028) 0.07
**Abnormal γ-GT**	122	86	<0.001	3.461 (2.138–5.603) <0.001
**Cholesterol – mg/Dl**	178.1±34.9	166.5±38.2	<0.001	0.991 (0.984–0.998) 0.01
**Insulin Resistance**	133	80	<0.001	1.688 (1.043–2.731) 0.03
**Histology** **at Biopsy**				
** Steatosis grade 0/1/2/3**	198/118/32/6	43/50/28/6	<0.001	1.275 (0.950–1.711) 0.10
** Grading 1 vs 2–3**	92/262	6/121	<0.001	5.767 (2.327–14.289) <0.001
**Chronic Hepatitis B**				
	**n = 125**	**n = 44**		
**Age** – **yrs**	37.0±13.6	49.4±10.8	<0.001	1.044 (1.003–1.088) 0.03
**Abnormal γ-GT**	17	18	<0.001	2.778 (1.041–7.414) 0.04
**Virological status**				
**HBe+/HBe−/HBe-Anti-HDV+**	26/94/5	2/33/9	<0.001	3.303 (1.229–8.877) 0.01
**Insulin Resistance**	105/20	22/22	<0.001	2.405 (1.038–6.197) 0.04
**Histology** **at Biopsy**				
** Steatosis grade 0/1/2/3**	88/30/7/0	14/25/3/2	<0.001	1.929 (0.978–3.804) 0.07
** Grading 1 vs 2–3**	50/75	3/41	<0.001	7.326 (1.690–31.754) 0.008

Data are given as mean±SD or as number of cases. Yrs, indicates years; γ-GT, γ-glutamiltranspherase.

**Table 3:** Diagnostic performance of GGT in predicting severe liver fibrosis.

AUC of variables for severe liver fibrosis prediction			
	AUC	C.I. 95%	p*
	**Non-alcoholic fatty liver disease**		
**γ-GT**	0.574	0.484–0.664	–
**Age**	0.739	0.654–0.834	0.009
**HOMA**	0.752	0.670–0.834	0.004
	**Chronic Hepatitis C**		
**γ-GT**	0.712	0.661–0.764	–
**Age**	0.659	0.606–0.712	0.16
**HOMA**	0.615	0.556–0.673	0.007
**Total cholesterol**	0.607	0.562–0.651	0.01
	**Chronic Hepatitis B**		
**γ-GT**	0.770	0.696–0.845	–
**Age**	0.759	0.683–0.836	0.81
**HOMA**	0.693	0.598–0.787	0.007
**PPV and NPV of abnormal/normal γ-GT for predicting severe liver fibrosis**			
	**PPV (%)**	**NPV (%)**	
**NAFLD**	**32.2**	**81.4**	
**CHC**	**41.3**	**84.9**	
**CHB**	**51.4**	**80.5**	

HOMA, homeostasis model assessment; γ-GT, γ-glutamiltranspherase; PPV, positive predictive value; NPV, negative predictive value. * p value of corresponding AUC compared to γ-GT AUC (pairwise comparison).

### Clinical and Laboratory Assessment

Clinical and anthropometric data were collected at the time of liver biopsy. Body mass index (BMI) was calculated on the basis of weight in kilograms and height in meters, and patients were classified as normal weight (BMI, 18.5–24.9 kg/m^2^), overweight (BMI, 25–29.9), or obese (BMI ≥30). A diagnosis of arterial hypertension was based on the following criteria: systolic blood pressure ≥130 mm Hg and/or diastolic blood pressure ≥85 mm Hg (measured three times in 30 minutes, in the sitting position and using a brachial sphygmomanometer), or use of blood-pressure-lowering agents. A diagnosis of type 2 diabetes was based on the revised criteria of the American Diabetes Association, using a value of fasting blood glucose ≥126 mg/dL on at least two occasions [23]. In patients with a previous diagnosis of type 2 diabetes, current therapy with insulin or oral hypoglycemic agents was documented.

**Table 4:** Univariate and Multivariate Analysis of Risk Factors Associated with Severe Fibrosis (F3–F4) in 504 Patients with Metabolic and Viral Chronic Liver Disease and normal GGT values, by Logistic Regression Analysis.

Variable	No Severe Fibrosis(F0–F2)	Severe Fibrosis(F3–F4)	Univariate Analysis *p* value	Multivariate Analysis OR (95% CI) *p* value
**Non-alcoholic Fatty Liver Disease**				
	**n = 79**	**n = 18**		
**Mean age** – **yrs**	42.5±13.1	54.6±11.1	0.01	1.070 (1.013–1.129) 0.01
**Male Gender**	12	7	0.02	2.132 (0.538–8.458) 0.28
**Mean body mass index** – **kg/m^2^**	29.6±4.5	31.8±3.9	0.05	1.028 (0.881–1.199) 0.72
**Insulin Resistance**	32	12	0.04	2.555 (0.671–9.727) 0.16
**Histology**				
** Lobular inflammation** 0/1/2/3	3/45/28/3	0/4/13/1	0.02	3.215 (1.150–8.987) 0.02
**Genotype 1 Chronic Hepatitis C**				
	**n = 232**	**n = 41**		
**Age** – **yrs**	50.9±12.3	57.3±8.5	0.002	1.034 (1.001–1.070) 0.04
**Male Gender**	100	31	<0.001	0.295 (0.129–0.673) 0.004
**Cholesterol – mg/Dl**	179.5±35.5	155.6±31.8	<0.001	0.981 (0.969–0.993) 0.003
**Insulin Resistance**	76	22	0.01	1.331 (0.613–2.889) 0.46
**Histology** **at Biopsy**				
** Steatosis grade 0/1/2/3**	148/71/11/2	18/16/6/1	0.02	1.605 (0.910–2.830) 0.10
** Grading 1 vs 2–3**	60/172	3/38	0.009	3.143 (0.873–11.316) 0.08
**Chronic Hepatitis B**				
	**n = 108**	**n = 26**		
**Age** – **yrs**	36.5±13.5	47.1±11.6	<0.001	1.037 (0.992–1.085) 0.10
**Virological status**				
**HBe+/HBe−/HBe-Anti-HDV+**	22/81/5	1/20/5	0.009	2.666 (0.879–8.087) 0.08
**Insulin Resistance**	19	10	0.02	1.383 (0.460–4.155) 0.56
**Histology** **at Biopsy**				
** Steatosis grade 0/1/2/3**	76/27/5/0	10/14/2/0	0.009	1.917 (0.831–4.425) 0.12
** Grading 1 vs 2–3**	46/62	1/25	<0.001	15.985 (1.894–134.892) 0.01

Data are given as mean ± SD or as number of cases. Yrs, indicates years; γ-GT, γ-glutamiltranspherase.

**Table 5:** Univariate and Multivariate Analysis of Risk Factors Associated with Severe Fibrosis (F3–F4) in 339 Patients with chronic liver disease (Chronic Hepatitis C, Non-alcoholic Fatty Liver Disease and Chronic Hepatitis B) and abnormal GGT values, by Logistic Regression Analysis.

Variable	No Severe Fibrosis(F0–F2)	Severe Fibrosis(F3–F4)	Univariate Analysis *p* value	Multivariate Analysis OR (95% CI) *p* value
**Non-alcoholic Fatty Liver Disease**				
	**n = 65**	**n = 31**		
**Mean age** – **yrs**	43.6±11.5	49.7±14.2	0.02	1.061 (1.015–1.111) 0.01
**Mean body mass index** – **kg/m^2^**	28.9±4.9	30.7±4.3	0.08	1.046 (0.911–1.201) 0.52
**Cholesterol – mg/Dl**	225.6±49.3	200.4±47.6	0.02	0.982 (0.968–0.996) 0.01
**Insulin Resistance**	25	24	<0.001	4.150 (1.079–15.970) 0.03
**Histology**				
** Lobular inflammation** 0/1/2/3	8/39/18/0	0/8/19/4	<0.001	6.824 (2.228–20.904) 0.001
**Genotype 1 Chronic Hepatitis C**				
	**n = 122**	**n = 86**		
**Age** – **yrs**	51.6±12.3	58.0±10.0	<0.001	1.049 (1.020–1.079) 0.001
**Insulin Resistance**	65/57	28/58	0.003	2.250 (1.211–4.181) 0.01
**Histology** **at Biopsy**				
** Grading 1 vs 2–3**	32/90	3/83	<0.001	8.970 (2.559–31.447) 0.001
**Chronic Hepatitis B**				
	**n = 17**	**n = 18**		
**Virological status**				
**HBe+/HBe−/HBe-Anti-HDV+**	4/13/0	1/13/4	0.05	13.757 (1.013–189.931) 0.04
**Insulin Resistance**	16/1	6/12	<0.001	3.096 (2.050–34.220) 0.01
**Histology** **at Biopsy**				
** Steatosis grade 0/1/2/3**	12/13/2/0	4/11/1/2	0.01	1.706 (0.316–9.209) 0.53

Data are given as mean ± SD or as number of cases. Yrs, indicates years; γ-GT, γ-glutamiltranspherase.

A 12-hour overnight fasting blood sample was drawn at the time of biopsy to determine serum levels of ALT, total cholesterol, triglycerides, plasma glucose concentration, insulin, and platelet count. Insulin resistance (IR) was determined by the homeostasis model assessment (HOMA) method, using the following equation [24]: insulin resistance (HOMA-IR) = fasting insulin (µU/mL) x fasting glucose (mmol/L)/22.5. HOMA-IR has been validated in comparison with the euglycemic/hyperinsulinemic clamp technique in both diabetic and non-diabetic patients [25]. HOMA-IR values >3 were considered to indicate IR [26].

Commercially available enzymatic colorimetric test was used for serum γ-GT assays. In accordance with the kit’s instructions, a serum γ-GT concentration of >36 IU/L in females, and >61 IU/L in males was considered the threshold value for identifying high levels of γ-GT.

HCV-infected individuals were tested at the time of biopsy for HCV-RNA using qualitative PCR (Cobas Amplicor HCV Test version 2.0; limit of detection: 50 IU/ml). HCVRNA-positive samples were quantified by Versant HCV-RNA 3.0 bDNA (Bayer Co. Tarrytown, NY, USA) expressed in IU/ml. Genotyping was performed by INNOLiPA (HCV II, Bayer HealthCare, Berkeley, CA, USA). Hepatitis B virus-infected patients were tested at the time of biopsy for HBs, HBeAg, anti-HBe and anti-HDV IgG, using commercial enzyme immunoassays (Dia Sorin, Saluggia, Italy). HBV-DNA was quantified by bDNA (Versant HBV 3.0, Siemens Medical Solutions Diagnostics Europe, Dublin, Ireland; range 357–17 857 000 IU/ml).

### Histology

Slides were coded and read by one pathologist (D.C.), who was unaware of the patient’s identity and history. Steatosis was assessed as the percentage of hepatocytes containing fat droplets (minimum 5%), and evaluated as a continuous variable. Steatosis was classified according to Kleiner classification as: absent <5%; mild 5%–33%; moderate >33%–66%; and severe >66%. In the NAFLD group, the Kleiner classification [27] was used to compute the NAFLD activity score (from 0 to 8, on a scale including separate scores for steatosis, lobular inflammation, and hepatocellular ballooning) and to stage fibrosis from 0 to 4. Biopsies of CHC and CHB patients were classified for grade and stage according to Scheuer’s system [28].

### Statistics

Continuous variables were summarized as mean±SD, and categorical variables as frequency and percentage. The Student’s t-test and chi-square test were used when appropriate. Multiple logistic regression analyses were done to identify factors independently associated with severe fibrosis in NAFLD, CHC and CHB patients considered separately, and in the sub-groups with high or normal γ-GT serum levels. As candidate risk factors, we selected age, gender, BMI, baseline ALT, normal or high γ-GT (only in the entire populations), triglycerides, total cholesterol, blood glucose, insulin, HOMA score, IR, diabetes, arterial hypertension, steatosis grade, lobular inflammation, ballooning, NAS score, and fibrosis. In these models, the dependent variable was severe fibrosis coded as 1 = present (F3–F4) versus 0 = absent (F0–F2).

Receiver operating characteristic (ROC) curves were applied to identify the area under ROC curve (AUC) of γ-GT and other variables to discriminate the presence of severe liver fibrosis.

Variables associated with the dependent variable on univariate analysis (probability threshold, p≤0.10) were included in the multivariate regression models. To avoid the effect of colinearity, diabetes, IR, HOMA score, blood glucose levels, and insulin levels, as well as NAS score and its components, were not included in the same multivariate model. Regression analyses were done using Proc Logistic, Proc Reg and subroutine in SAS (SAS Institute, Inc., Cary, North Carolina, U.S.A.) [29].

## Results

### Patient Characteristics and Histology

Abnormal values of serum γ-GT levels were observed in 49.7%, 43.2% and 20.7% of NAFLD, G1 CHC and CHB patients, respectively. The baseline characteristics of the 843 patients according to the etiology of liver disease, and to the presence of normal or high γ-GT serum levels are shown in Table 1 and in Supplemental Table S1. Overall, patients with abnormal γ-GT serum levels had higher ALT serum levels and, considering liver histological features, a more severe liver disease, in term of fibrosis in NAFLD, and of steatosis, inflammatory activity and fibrosis in both CHC and CHB (Figure 1). In addition, patients with abnormal γ-GT had more frequent metabolic alterations, namely higher cholesterol and triglycerides levels in NAFLD, higher triglycerides levels and a higher prevalence of IR and diabetes in CHC, and higher cholesterol and triglycerides and a higher prevalence of IR and hypertension in CHB.

Due to the evidence of a higher prevalence of IR in CHC and CHB patients with abnormal γ-GT values compared to their counterpart, in both CHC and CHB groups we tested if this association was maintained at multivariate analysis. We found that, after confirming older age (OR 1.025, CI 1.006–1.045, p = 0.01 for CHC), and higher BMI (OR 1.171, CI 1.105–1.242, p<0.001 for CHC; OR 1.211, CI 1.071–1.369, p = 0.002 for CHB) as independent predictors of IR, abnormal γ-GT values remained independently associated with IR in CHC patients only (OR 1.861, CI 1.228–2.821, p = 0.003). In this group of patients the AUC of γ-GT values for IR prediction was 0.652 (C.I. 0.603–0.705), compared to 0.609 (C.I. 0.603–0.705) (p = 0.22) of age, and 0.689 (C.I. 0.642–0.736) (p = 0.27) of BMI. Finally, the positive predictive value (PPV) of abnormal γ-GT for IR prediction was 55.2%, while the negative predictive value (NPV) of normal γ-GT was 64.1%.

### Factors Associated with Severe Liver Fibrosis in the Entire Cohort of Patients with CLD

Severe fibrosis (F2–F4) was independently associated with abnormal γGT values in NAFLD (OR 2.711, CI 1.120–6.564, p = 0.02), G1 CHC (OR 3.461, CI 2.138–5.603, p<0.001), and CHB (OR 2.778, CI 1.042–7.414, p = 0.04) patients, together with other well known risk factors for fibrosis like older age, IR and lobular inflammation in NAFLD (Table 2 up), older age, lower cholesterol, IR and moderate-severe necroinflammatory activity in G1 CHC (Table 2 medium), and older age, virological status, IR and moderate-severe necroinflammatory activity in CHB (Table 2 bottom) patients. The AUC of the entire models were 0.830 (C.I. 0.769–0.880) for NAFLD, 0.798 (C.I. 0.759–0.833) for CHC, and 0.855 (C.I. 0.793–0.904) for CHB. The PPV and NPV of abnormal and normal γGT values, respectively, for severe fibrosis prediction, were reported in table 3.

Finally, when replacing in the model γGT as categorical variable, with γGT as continuous variable, the latter remained significantly associated with severe fibrosis in NAFLD (OR 1.003, CI 1.001–1.006, p = 0.04), G1 CHC (OR 1.004, CI 1.001–1.007, p = 0.004), and CHB patients (OR 1.022, CI 1.005–1.039, p = 0.01). The AUC of γGT values for severe fibrosis prediction, compared to those of the other variables, were reported in table 3.

### Factors Associated with Liver Fibrosis in Patients with CLD and with Normal γGT Serum Levels

In patients with normal γGT values, severe fibrosis was independently associated with older age (OR 1.070, CI 1.013–1.129, p = 0.01) and lobular inflammation (OR 3.215, CI 1.150–8.987, p = 0.02) in NAFLD (Table 4 up), older age (OR 1.034, CI 1.001–1.070, p = 0.04), no male gender (OR 0.295, CI 0.129–0.673, p = 0.004), and lower cholesterol levels (OR 0.981, CI 0.969–0.993, p = 0.003) in G1 CHC (Table 4 medium), and with only moderate-severe liver necroinflammatroy activity (OR 15.985, CI 1.894–134.893, p = 0.01) in CHB (Table 4 bottom) patients.

### Factors Associated with Liver Fibrosis in Patients with CLD and with Abnormal γGT Serum Levels

In patients with abnormal γGT values, severe fibrosis was independently associated with older age (OR 1.061, CI 1.015–1.111, p = 0.01), lower cholesterol levels (OR 0.982, CI 0.968–0.996, p = 0.01), IR (OR 4.150, CI 1.079–15.970, p = 0.03), and lobular inflammation (OR 6.824, CI 2.228–20.904, p = 0.001) in NAFLD (Table 5 up), with older age (OR 1.049, CI 1.020–1.079, p = 0.001), IR (OR 2.250, CI 1.211–4.181, p = 0.01), and moderate-severe liver necroinflammatory activity (OR 8.970, CI 2.559–31.447, p = 0.001) in G1 CHC (Table 5 medium), and with virological status (OR 13.757, CI 1.013–189.931, p = 0.04), and IR (OR 3.096, CI 2.050–34.220, p = 0.01) in CHB (Table 5 bottom) patients.

## Discussion

In a large cohort of patients with an histological diagnosis of CLD, other than to confirm the independent association between higher γ-GT serum levels and severity of liver fibrosis, we showed that, IR is independently associated with severe fibrosis only in patients with abnormal γ-GT values, without differences according to the metabolic (NAFLD) or viral (HCV or HBV) etiology of the underlying liver disease.

In patients with CLD the severity of liver fibrosis is the strongest predictor of liver related morbidity and mortality, being, in a context of genetic predisposition, IR and liver necroinflammation two of the most important factors associated with the severity and the progression of liver disease [3]–[8]. Specifically, IR has been clearly associated with fibrosis severity in NAFLD and CHC patients, while contrasting data exist in CHB. In our study, we confirmed both IR and liver necroinflammation as independently related to the severity of liver fibrosis in patients with NAFLD and CHC, adding data pros this association in CHB.

In our study, we identified in high γ-GT serum levels a risk factor for severe fibrosis. This feature is in accord not only to when highlighted by other authors in NASH [19], and to the evidences about an association between γ-GT serum levels reduction and regression of liver damage in NAFLD patients after bariatric surgery [20], but also to different studies showing an independent link between higher γ-GT serum levels and severity of fibrosis in both CHC [21] and CHB patients [22], and potentially to the role of γ-GT serum levels as predictor of all-cause deaths, including liver-related death [20]. Interestingly, in our cohort, the presence of abnormal γ-GT, had a NPV of more than 80% for rule-out severe fibrosis in all patients, independently of the aetiology of liver disease. This data is encouraging, even if, in clinical practice, we are not confident to suggest to use γ-GT as a single test to predict or rule-out fibrosis severity, but as a potential useful component of noninvasive scores.

Considering therefore the association between γ-GT and liver disease severity, the evidences about a link between γ-GT values and metabolic alterations, like visceral obesity, diabetes, etc, in both cross-sectional and prospective studies [15], [16], and also observed in our population, and the sometime contrasting evidences on the role of IR in liver disease severity [4], [9], [10] and in NAFLD treatment [11], [12], we speculated that discriminating NAFLD patients according to normal or abnormal γ-GT values, we can identify different patterns of liver disease where metabolic alterations, and in particular IR, could have a different impact. In this line, for the first time to our knowledge, we found that, while classical host and viral risk factors were confirmed as independently linked to the severity of liver fibrosis in NAFLD, CHC and CHB patients with high or normal γ-GT values, by contrast IR remained significantly associated with liver fibrosis only in patients with abnormal γ-GT levels nevertheless similar HOMA values were observed in those with normal or high γ-GT levels, and independently of the etiology of the underlying liver disease.

Although this study was not designed to clarify this interesting finding, a few hypotheses may be put forward according to the literature. A German cohort study found that hepatic steatosis is a significant predictor of all-cause and cardiac-related mortality in men only when it was associated with high γ-GT levels [30]. Another population Italian study also showed that, considering a non-invasive index for steatosis diagnosis (fatty liver index) composed by different metabolic traits including γ-GT, only γ-GT was predictive of all-cause mortality in a context of presence of other metabolic alterations [31]. Finally it has recently been described that serum γ-GT levels have shared genetic determinants which may include the b-2 adrenergic receptor gene [32] and loci interacting with cardiometabolic disease alterations [33]. Overall these data could suggest that γ-GT, in a context of metabolic disturbances, may reflect genetic and metabolic determinants which are associated with poorer outcomes. In this line, according to these data and to our findings on patients with CLD due to metabolic or viral ones, it should be possible to speculate that high γ-GT levels identify a group of liver patients whit a metabolic milieu probably also influenced by genetic background, and able to participate in liver damage affecting the severity of disease. Instead normal γ-GT levels probably identify another group of patients with CLD where metabolic alterations, and in particular IR, even if present, have not a dominant role in affecting disease severity. By contrast in these patients conventional risk factors, like liver inflammation, are the main determinant of disease severity, suggesting a potential dominant role of inflammatory genetic background. In this line we recently reported that IL28B polymorphisms are able to affect severity of liver inflammation in NAFLD patients independently of metabolic alterations [34].

From a clinical standpoint, the identification of these two different patterns of CLD according to γ-GT values, could be useful in the management of these patients. In fact overall our results might also suggest to evaluate if γ-GT can identify NAFLD, CHC and CHB patients at different liver disease progression risk and with different natural history, or where the correction of IR is of further help. In this line our data could help to better understand because: 1) IR is associated with fibrosis severity in a great proportion but not in all studies on CHC patients; and 2) very contrasting results exist about the association between IR and severity of fibrosis in CHB. In addition, considering NAFLD, even if is well known that lifestyle correction is the gold standard treatment for these patients [35], conflicting results exist about pharmacologic therapy. In particular literature data showed that insulin sensitizer [11] and antioxidant drugs [12] could improve liver histological damage, but only in a proportion of NAFLD patients. The discrimination of NAFLD patients according to γ-GT values could therefore help to distinguish patients more sensitive to an approach (lifestyle and/or pharmacological) aimed to correct metabolic dysfunctions, to patients more sensitive to drugs acting on oxidative stress and liver inflammation. Our hypotheses would seem to suggest using γ-GT as a way of stratify NAFLD patients in intervention trials, even if, clearly, further studies must be done, in diverse settings and with larger cohorts of patients, before applying this method in clinical practice.

The main limitation of this study lies in its cross-sectional nature, making it impossible to dissect the temporal relation among γ-GT values, liver fibrosis, and both metabolic and liver inflammatory findings in patients with CLD. A further methodological question is the potentially limited external validity of the results for different populations and settings. Our study included a cohort of Italian patients enrolled at a tertiary care center, who may be different from the majority of cases of CLD in the general population. In particular we enrolled patients with abnormal ALT levels and, for CHC and CHB, with a high viral load; therefore our results could be applied only to this setting of patients and not to those with a milder liver disease. An hidden alcohol abuse, and lack of data on genetic polymorphisms affecting CLD severity and γ-GT serum levels may also have affected interpretation of the results.

In conclusion, we confirmed γ-GT serum levels as a marker of fibrosis in patients with CLD useful to be included in noninvasive scores, and observed that IR is independently linked to liver fibrosis only in patients with abnormal γ-GT values, without differences according to the metabolic (NAFLD) or viral (HCV or HBV) etiology of the underlying liver disease, and suggesting a role of γ-GT as a marker of metabolic-induced liver damage. These data need further validation in independent, large scale studies, potentially representing an useful tools for the clinical and pharmacologic management of patients with CLD.

## Supporting Information

Table S1 Demographic, Clinical, Biochemical, and Histological Features of 843 Patients with Metabolic and Viral Chronic Liver Diseases, According to the Etiology and to γ-glutamiltranspherase Serum Levels.
(DOC)
