# Supplementary material for: Serum γ-glutamyl Transferase Levels, Insulin Resistance and Liver Fibrosis in Patients with Chronic Liver Diseases
Source: PLoS One. 2012 Dec 5;7(12):e51165. doi: 10.1371/journal.pone.0051165 (PMC3515567; doi:10.1371/journal.pone.0051165)
Supplement: Table S1 — Demographic, Clinical, Biochemical, and Histological Features of 843 Patients with Metabolic and Viral Chronic Liver Diseases, According to the Etiology and to γ-glutamiltranspherase Serum Levels. (DOC) [file pone.0051165.s001.doc]

**Table S1. Demographic, Clinical, Biochemical, and Histological Features of 843 Patients with Metabolic and Viral Chronic Liver Diseases, According to the Etiology and to γ-glutamiltranspherase Serum Levels**.

| **Variable** | **NAFLD**  **N=193** | |  | **Genotype 1 Chronic Hepatitis C**  **N=481** | |  | **Chronic Hepatitis B**  **N=169** | |  |
| --- | --- | --- | --- | --- | --- | --- | --- | --- | --- |
|  | **normal γGT**  (n=97) | **abnormal γGT**  (n=96) | **P**  **value** | **normal γGT**  **n=273** | **abnormal γGT**  **n=208** | **P**  **value** | **normal γGT**  **n=134** | **abnormal γGT**  **n=35** | **P value** |
| **Mean age** – **yrs** | 44.7 ± 13.6 | 46.7 ± 13.2 | 0.31 | 51.9 ± 12.1 | 54.2 ± 11.8 | 0.03 | 38.6 ± 13.7 | 46.7 ± 13.1 | 0.002 |
| **Male Gender** | 78 | 50 | <0.001 | 131 | 111 | 0.24 | 102 | 21 | 0.05 |
| **Mean body mass index** – **kg/m^2^** | 30.0 ± 4.5 | 29.5 ± 4.8 | 0.42 | 26.2 ± 4.2 | 26.7 ± 4.1 | 0.22 | 25.4 ± 3.2 | 26.0 ± 3.3 | 0.37 |
| **Body mass Index** – **kg/m^2^**  <25  25-29.9  ≥30 | 10  46  41 | 17  38  41 | 0.27 | 103  129  41 | 63  107  38 | 0.21 | 57  61  16 | 14  16  5 | 0.92 |
| **Alanine aminotransferase – IU/L** | 70.0 ± 56.7 | 89.2 ± 52.9 | 0.01 | 61.7 ± 38.2 | 128.4 ± 128.8 | <0.001 | 93.7 ± 94.9 | 138.5 ± 99.5 | 0.01 |
| **γ-glutamiltranspherase –IU/L** | 32.8 ± 13.7 | 155.9 ± 143.2 | <0.001 | 27.5 ± 13.4 | 135.4 ± 155.5 | <0.001 | 28.5 ± 13.3 | 83.5 ± 46.9 | <0.001 |
| **Cholesterol – mg/Dl** | 192.8 ± 42.0 | 217.0 ± 49.9 | <0.001 | 175.9 ± 36.0 | 173.8 ± 36.4 | 0.53 | 185.2 ± 39.1 | 211.7 ± 57.7 | 0.01 |
| **Triglycerides – mg/dL** | 136.4 ± 69.8 | 161.9 ± 83.1 | 0.02 | 88.2 ± 40.0 | 107.2 ± 49.1 | <0.001 | 96.7 ± 33.2 | 117.2 ± 66.6 | 0.01 |
| **Blood glucose – mg/dL** | 97.4 ± 23.0 | 101.1 ± 37.8 | 0.41 | 92.9 ± 25.4 | 103.7 ± 42.3 | 0.001 | 88.5 ± 16.8 | 98.0 ± 28.5 | 0.01 |
| **Insulin – μU/mL** | 16.3 ± 18.5 | 18.2 ± 11.8 | 0.20 | 12.3 ± 8.9 | 14.1 ± 7.5 | 0.02 | 10.0 ± 6.9 | 12.0 ± 8.0 | 0.15 |
| **HOMA-score** | 4.05 ± 2.95 | 4.73 ± 3.93 | 0.18 | 2.97 ± 2.23 | 3.56 ± 2.64 | 0.001 | 2.21 ± 1.60 | 3.02 ± 2.37 | 0.01 |
| **Insulin Resistance** | 44 | 49 | 0.43 | 98 | 115 | <0.001 | 29 | 13 | 0.05 |
| **Type 2 diabetes** | 17 | 16 | 0.84 | 29 | 40 | 0.008 | 3 | 3 | 0.07 |
| **Arterial hypertension** | 18 | 27 | 0.11 | 59 | 53 | 0.32 | 4 | 4 | 0.03 |
| **LOG HCVRNA** | - | - | - | 5.6 ± 0.7 | 5.7 ± 0.6 | 0.28 | - | - | - |
| **LOG HBVDNA** | - | - | - | - | - | - | 4.9 ± 1.5 | 5.0 ± 1.7 | 0.73 |
| **Virological Status**  **HBeAg+/HBeAg-/HBeAg-Anti-HDV+** | - | - | - | - | - | - | 23/101/10 | 526/4 | 0.71 |
| **Histology**  **NAFLD activity score (NAS)**  1-2  3-4  5-8  **Kleiner Lobular inflammation**  0  1  2  3  **Kleiner Steatosis grade**  0 (<5%)  1 (5%-33%)  2 (>33%-66%)  3 (>66%)  **Hepatocellular ballooning**  0  1  2  **Kleiner Stage of Fibrosis**  **0**  **1**  **2**  **3**  **4**  **Scheuer Necroinflammatory Activity**  **0**  **1**  **2**  **3**  **Scheuer Stage of Fibrosis**  **0**  **1**  **2**  **3**  **4** | 6 (6.1)  25 (25.8)  66 (68.1)  3 (3.0)  49 (50.5)  41 (42.3)  4 (4.2)    0 (0)  30(30.9)  35 (36.1)  32 (33.0)  8 (8.2)  36 (37.1)  53 (54.7)  22 (22.7)  30 (30.9)  28 (28.9)  15 (15.5)  2 (2.0)  -  -  -  -  -  -  -  -  - | 12 (12.5)  52 (54.2)  32 (33.3)  8 (8.3)  47 (48.9)  37 (38.6)  4 (4.2)  0 (0) 39 (40.6)  26 (27.1)  31 (32.3)  12 (12.5)  37 (38.5)  47 (49.0)  25 (26.0)  22 (22.9)  18 (18.7)  20 (20.8)  11 (11.6)  -  -  -  -  -  -  -  -  - | 0.27  0.47  0.28  0.51  0.04  -  - | -  -  -  -  -  -  -  166 (60.8)  87 (31.9)  17 (6.2)  3 (1.1)  -  -  -  -  -  -  -  -  1 (0.3)  62 (22.7)  163 (59.7)  47 (17.3)  22 (8)  79 (29.8)  131 (48)  29 (10.6)  12 (4.5) | -  -  -  -  -  -  -  75 (36)  81 (38.9)  43 (20.7)  9 (4.4)  -  -  -  -  -  -  -  -  1 (0.4)  34 (16.4)  93 (44.7)  80 (38.5)  11 (5.3)  42 (20.2)  69 (33.2)  48 (23)  38 (18.3) | -  -  <0.001  -  -  <0.001  <0.001 | -  -  -  -  -  -  -  86 (64.2)  41 (30.6)  7 (5.2)  0 (0)  -  -  -  -  -  -  -  -  0 (0)  47 (35)  52 (38.8)  35 (26.2)  7 (5.2)  46 (34.3)  55 (41.1)  18 (13.4)  8 (6) | -  -  -  -  -  -  -  16 (45.7)  14 (40)  3 (8.6)  2 (5.7)  -  -  -  -  -  -  -  -  0 (0)  6 (17.1)  11 (31.4)  18 (51.5)  1 (2.9)  11 (31.5)  5 (14.4)  8 (22.6)  10 (28.6) | -  -  -  -  -  -  -  0.01  -  -  0.01  0.001 |

Data are given as mean ± SD or as number of cases. Yrs, indicates years; HOMA, homeostasis model assessment; hepatitis C virus ribonucleic acid; HBV-DNA, hepatitis B virus deoxyribonucleic acid.
